# Conductive vial electromembrane extraction – Principles and practical operation

**DOI:** 10.1002/ansa.202200065

**Published:** 2023-07-21

**Authors:** Maria Schüller, Frederik André Hansen, Tonje Gottenberg Skaalvik, Stig Pedersen‐Bjergaard

**Affiliations:** ^1^ Department of Pharmacy University of Oslo, Blindern Oslo Norway; ^2^ Department of Clinical Pharmacology St. Olav University Hospital Trondheim Norway; ^3^ Department of Pharmacy, Faculty of Health and Medical Sciences University of Copenhagen Copenhagen Denmark

**Keywords:** conductive vial electromembrane extraction, microextraction, sample preparation, tutorial

## Abstract

Electromembrane extraction (EME) is a microextraction technique where charged analytes are extracted from an aqueous sample solution, through a liquid membrane, and into an aqueous acceptor, under the influence of an external electric field. The liquid membrane is a few microliters of organic solvent immobilized in a polymeric support membrane. EME is a green technique and provides high selectivity. The selectivity is controlled by the direction and magnitude of the electric field, the chemical composition of the liquid membrane and the pH. Recently, commercial prototype equipment for EME was launched based on the use of conductive vials, and interest in EME is expected to increase. The current article is a tutorial and discusses the principle and practical work with EME. The practical information is related to the commercial prototype equipment but is valid also for other technical configurations of EME. The tutorial is intended to give readers a fundamental understanding of EME, which is required for method development and operation, and for avoiding common pitfalls.

## 1 INTRODUCTION

Electromembrane extraction (EME) is a microextraction technique introduced in 2006.^1^ The concept is unique by utilizing an electric field to isolate target compounds (analytes) from complex samples, through a liquid membrane of a few microliters of organic solvent. Since its introduction, EME has gained substantial attention in the scientific literature, and the concept has been discussed in more than 400 published papers. Reasons for this interest include EME being a simple principle providing fast and selective extractions performed in a single step, while also representing green analytical chemistry. To date, EME has been realized in various homemade technical formats, including hollow‐fibre, 96‐well and microfluidic systems. Commercial equipment has, however, not been available, being a significant shortcoming for the wider adoption of the technique. Commercial equipment is now in the final stages of development by Extraction Technologies Norway AS and is expected to become available during 2023. The launch is expected to increase interest in EME. In the current tutorial, we, therefore, discuss the principles and practical aspects of EME and provide representative examples of established applications. The tutorial is aligned with the prototype format, but all considerations discussed are generally valid for EME. For detailed discussions on EME theory and an overview of the scientific literature, we refer elsewhere.^2^, ^3^, ^4^, ^5^, ^6^, ^7^ We hope this tutorial may help and inspire new EME users in their development of EME. Common pitfalls are covered systematically and comprehensively for the first time, and this information is highly important for future scientists working with EME.

## 2 PRINCIPLE

**FIGURE 1:**
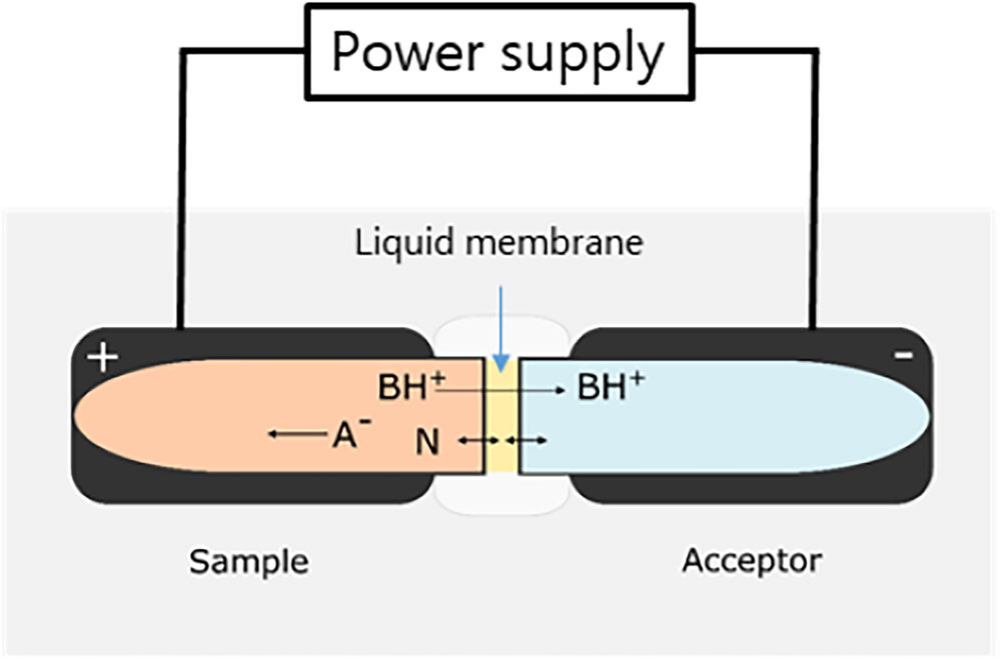
Principle of conductive vial electromembrane extraction (EME) principle. BH^+^ represents a protonated base, A^−^ is deprotonated acid, and N is a neutral molecule. The EME cell comprises two conductive vials, a flat circular support membrane impregnated with membrane solvent and union holding the supported liquid membrane between the two vials.

The principle for the commercial EME format with conductive vials is illustrated in Figure 1. The aqueous sample solution (in the sample vial) is separated from a clean acceptor solution (in the acceptor vial) by a thin liquid membrane. The liquid membrane is a microliter volume of organic solvent immobilized in the pores of a thin and porous support membrane (∼100 µm), typically made of polypropylene. The liquid membrane is robust and held in position by capillary forces, handling strong shear force without losing integrity. In addition, the volume of organic solvent is typically in the low‐µL range, which makes the consumption negligible from a green chemistry perspective. Extraction progresses by substances partitioning into the liquid membrane and further by transfer to the acceptor. After extraction, the acceptor is collected and analysed directly with an instrumental method such as liquid chromatography, without the need for any further filtration, evaporation and reconstitution. The volumes of sample solution and acceptor can be equal, but operating with less acceptor is also feasible if enrichment is desired. The extraction may be driven by a pH gradient across the liquid membrane, in which case it is termed liquid‐phase microextraction (LPME). However, this mode of extraction is based on passive diffusion and is thus rather slow. In EME, the extraction process is augmented by applying an electric field (direct current) across the liquid membrane, which improves the kinetics considerably by actively transporting ionic substances in the electric field. The applied voltage in EME is typically in the range from 10 to 100 V, and the electric field (V/cm) is relatively strong due to the short distance between electrodes. During EME, a small electric current (microampere (µA) level) is generated as ions transfer across the liquid membrane. Recording this extraction current may be used for general process monitoring, as discussed below.

EME provides a high degree of selectivity and sample clean‐up, as neutral substances are uninfluenced by the electric field. The direction of the electric field controls if positive (cations) or negative (anions) ions are extracted and the selectivity is to a large extent determined by the chemical composition of the liquid membrane and by the magnitude of the electric field (extraction potential). EME is therefore excellent for the clean‐up of matrix components. Using EME, extracts from blood plasma and serum are free of proteins, phospholipids, salts and most endogenous metabolites.^8^, ^9^ EME has previously been applied for the extraction of a wide variety of analytes, including pharmaceuticals,^10^, ^11^ endogenous metabolites,^12^, ^13^ peptides,^14^, ^15^ heavy metal ions,^16^, ^17^salt ions^18^, ^19^ and environmental contaminants.^20^, ^21^ EME is commonly used to transfer analytes from a complex sample matrix into a clean acceptor solution but may also be applied to remove matrix substances present at high levels, such as excess fluorescent dye or surfactants.^22^, ^23^

## 3 EQUIPMENT

**FIGURE 2:**
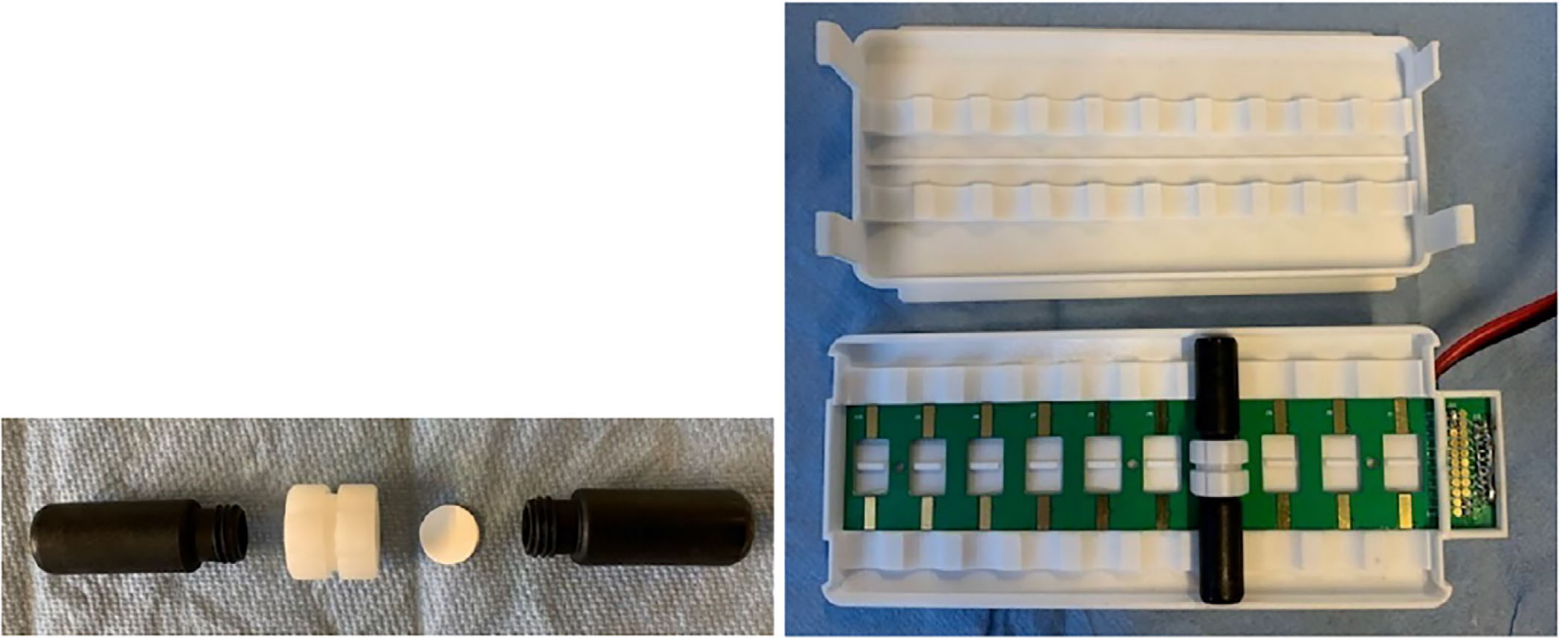
Left: sample vial, support membrane union, circular support membrane and acceptor vial; right: 10‐position holder with 1 extraction cell.

The EME equipment comprises 5 different components, namely the extraction cells (Figures 1 and 2), a 10‐position holder for the extraction cells (Figure 2), an agitator, an external power supply and an ammeter. Here, each extraction cell comprises a sample vial, an acceptor vial, a circular sheet of support membrane and a union holding the supported membrane (Figure 2). The sample and acceptor vials are made from a conducting polymer material, serving both as vials and as electrodes. The vials are currently available in two volumes, namely 200 and 600 µL. After loading the sample solution, acceptor and liquid membrane, the sample vial and acceptor vial are attached to the support membrane union, and the extraction cell is placed in the 10‐position holder. For currently available versions of the device, the liquid membranes are loaded manually on to the support filters, and 10 samples can be extracted simultaneously. The sample and acceptor vials are connected to the external power supply via the 10‐position holder. The 10‐position holder is also fixed to an agitation system, allowing for the horizontal agitation of the extraction cells to promote mass transfer. An ammeter is used to measure the total current through the extraction cells and should be used as a diagnostic tool to check the intactness of the liquid membrane. In the case of membrane failure in one of the extraction cells, the electric resistance is significantly reduced, and excessive current is observed. As the extraction cells are coupled in parallel, membrane failure in one extraction cell does not affect the other extraction cells. In the final commercial equipment, the current will be measured for each extraction cell, and this will increase operational flexibility and control. EME has been performed in laboratory‐built 96‐well systems^24^ for high‐throughput operation, and commercial products in this format are expected soon. The final commercial system, which will be available during 2023, will be controlled and operated with tailor‐made software.

## 4 METHOD DEVELOPMENT

The main operational parameters in EME include the composition of the liquid membrane, the choice of sample diluent, the choice of the acceptor, the extraction potential, the agitation rate and the extraction time. The selection of the liquid membrane is normally the first step of method development. The optimal values for the operational parameters may be influenced by the composition of the sample matrix; therefore, optimization is better performed with spiked matrix samples.

### 4.1 Liquid membrane

The solvent selected as the liquid membrane should comply with the following criteria:

- Solubility in water should be less than 0.5 mg/mL.
- Octanol–water partition coefficient (log *P*) should not be less than 3.0.
- The boiling point should not be less than 150–200°C

**FIGURE 3:**
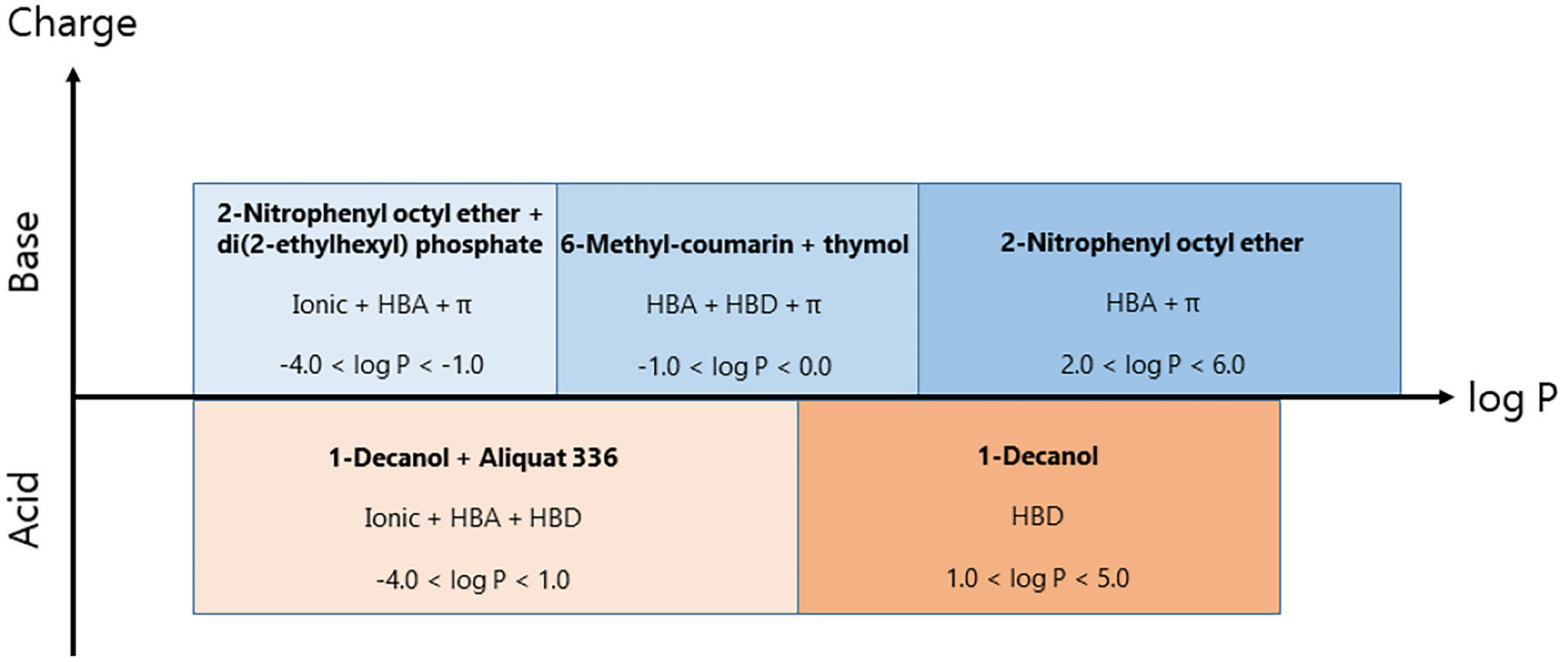
Recommended liquid membranes for electromembrane extraction (EME). HBA, hydrogen bond acceptor; HBD, hydrogen bond donor.

For the extraction of organic bases and acids less than 1000 Daltons, the selection of liquid membrane may be guided by Figure 3. Basic analytes are protonated during EME, and they are transferred into the liquid membrane as cations. For this transfer to be significant, electric potential is required. In addition, proper solvation of the protonated analyte (solute) in the liquid membrane (solvent) is required, facilitated by strong solute–solvent interactions. Strong solute–solvent interactions are mainly hydrogen bonding, cation‐π and ionic interactions. Extraction of basic analytes with high hydrophobicity (log *P* > 2.0) is facilitated by hydrogen bonding and cation‐π interactions; therefore, 2‐nitrophenyl octyl ether (NPOE) is the first choice as membrane solvent. The protonated basic analytes are hydrogen bond donors (HBDs) and π electron acceptors, and NPOE serves as a hydrogen bond acceptor (HBA) and π electron donor (π). For bases, the extraction window of NPOE is 2.0 < log *P* < 6.0, which means that compounds in this log *P* range are normally extracted with high recovery using NPOE.

NPOE is a perfect liquid membrane in EME for several reasons. The water solubility is extremely low (0.0008 mg/mL), and leakage into the sample solution and acceptor is minimal. The liquid membrane of NPOE is therefore stable during extraction. Second, liquid membranes of NPOE are stable in contact with biological fluids and other complex sample solutions. Therefore, NPOE can be used for extraction from a variety of biological samples. NPOE is non‐volatile, and the evaporation of the liquid membrane during EME preparations is negligible. Lastly, the electrical resistance of NPOE is high, and therefore, NPOE can be operated at potentials exceeding 100 V without further practical implications related to the electrolysis in the sample solution and acceptor (discussed further in Section 6.1). It should be noted that NPOE is generally more expensive than traditional liquid–liquid extraction solvents; this, however, is partly compensated by the small volumes used in EME.

For more hydrophilic bases (log *P* < 2.0), mass transfer is limited with NPOE because this liquid membrane is too hydrophobic (log *P* = 4.86). For extraction of bases in the range 0.0 < log *P* < 2.0, 2‐undecanone or tri(pentyl) phosphate may be alternative liquid membranes with lower hydrophobicity. The deep eutectic solvent composed of 6‐methyl‐coumarin and thymol (1:2 w/w) may also be relevant within the log *P* range from −1.0 to 2.0. Extraction of bases with log *P* < −1.0 normally requires the addition of an ionic carrier to the liquid membrane, to facilitate ionic solute–solvent interactions. The preferred ionic carrier is di(2‐ethylhexyl) phosphate (DEHP), and this is added to NPOE in concentrations of 1–10% w/w. Several other liquid membranes have also been tested and could be considered in method development.^12^

Acidic analytes are extracted as anions. For acids with log *P* > 1, hydrogen bond interactions are dominant for analyte solvation. Deprotonated acids serve as HBAs, and efficient liquid membranes are organic solvents with HBD properties. Higher alcohols such as 1‐nonanol and 1‐decanol are potential liquid membranes. 1‐Octanol is often used, but water solubility is significant (0.5 mg/mL), and the EME system is not sufficiently stable from the authors' point of view. For acids with log *P* < 1.0, ionic solute–solvent interactions are required, and Aliquat 336 has been proposed as an ionic carrier. Aliquat 336 is a technical mixture of alkylated quaternary ammonium salts, with methyl‐trioctylammonium chloride as the main constituent. The content of Aliquat 336 should be low (0.5–2.0% w/w) to avoid excessive extraction current. 1‐Nonanol and 1‐decanol are currently recommended membrane solvents. However, liquid membranes reported for hydrophilic acids are of limited stability and performance, and more stable alternatives are under development.

EME has also been used for the extraction of inorganic ions and small peptides. Liquid membranes used for these applications can be found in the literature and are not discussed further in this tutorial.^3^, ^15^, ^25^, ^26^

### 4.2 Sample diluent and acceptor

For efficient EME, the target analyte should be fully charged in the sample and acceptor solution. Occasionally, EME can be performed directly from the crude sample, after the addition of appropriate internal standards. This is, however, not generally recommended due to the lack of pH control. Sample diluents, ideally in the form of buffers, should be used for appropriate pH adjustment. Inorganic or polar organic acids and bases are recommended, due to their low affinity for the liquid membrane. Typical chemicals are H_3_PO_4_, NaH_2_PO_4_, Na_2_HPO_4_, Na_3_PO_4_, HCOOH, NaHCOO, NH_3_, NaHCO_3_ and Na_2_CO_3_. For the selection of the acceptor solution, compatibility with the subsequent instrumental method should be considered. In cases where acceptors are analysed by LC–MS, volatile components should be selected, such as dilute formic acid or ammonia.

For basic analytes, pH in the sample and acceptor solution should preferably be two to three units below the p*K*_a_ value of the analyte. Under such conditions, the analyte is fully ionized, and the extraction is only facilitated by the extraction potential. If the pH in the sample solution is closer to p*K*_a_, more analyte molecules are neutral, and part of the mass transfer is facilitated by passive diffusion, where the extraction is a mix of EME and LPME. In a similar way, for EME of acidic analytes, sample and acceptor pH should preferably be two to three pH units above p*K*_a_.

### 4.3 Extraction potential, agitation and time

**FIGURE 4:**
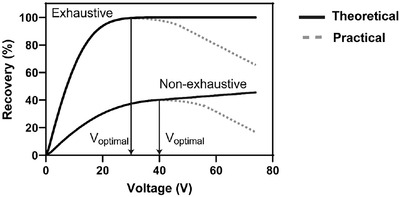
Illustration of theoretical and practical effects of extraction potential on extraction recovery for exhaustive and non‐exhaustive systems.

The recovery and extraction time are related to the extraction potential. Recovery is increasing with increasing extraction potential, whereas the time to reach steady state conditions (extraction time for maximum recovery) is decreasing with increasing extraction potential. At a certain potential, however, there is no additional effect of increasing the voltage (Figure 4), and extractions should be conducted at this potential (or below). With NPOE as liquid membrane, the optimal extraction potential is often in the range 50–150 V. With other liquid membranes, the optimal extraction potential may be much lower. We recommend measuring the current in each extraction cell during EME, and this should not exceed 50 µA per cell. Operation with high extraction current may result in unstable EME systems. The extraction current is proportional to the extraction potential according to the Ohms law, and therefore, current is reduced by using a lower extraction potential.

The main voltage drop in the EME system is across the liquid membrane, whereas the electric field is relatively weak in the bulk aqueous sample solution. Mass transfer in the sample solution is therefore mainly by convection and diffusion, and agitation is important. Typical agitation rates are in the range 750–1000 rotations per minute (rpm). With less agitation, mass transfer is reduced, whereas at higher agitation rates, the EME system tends to be more unstable. The optimal agitation rate depends on the volume of the vials, and this should be considered during method development.

Extraction recovery in EME is increasing with time, until a certain point where the system enters steady state conditions; here, the extraction should be terminated. In theory, if the extraction current is low and the system is well buffered, the extraction could be extended beyond the point of steady‐state conditions. However, some EME systems (mainly liquid membrane suitable for hydrophilic analytes) may become unstable for very long extractions and should be terminated prior to this point. Typical extraction times are in the range of 5–30 min, depending on the type of analyte, the experimental conditions and the size of the conductive vials. Current vials are 200 and 600 µL, and kinetics are often faster with the smaller vials. Extraction potential, agitation rate and extraction time are typically set based on experimental optimization experiments.

## 5 EXTRACTION PROCEDURE

Handling the conductive vials and support membranes before the extraction should be done with care. As the electric field is coupled through the vials, these should preferably be handled with laboratory gloves. The same holds for the support membranes. In the current prototype EME equipment, the support membranes are delivered separately, and as the first step, the support membranes are inserted into the support membrane unions. In the final commercial equipment, this first step will be completed by the manufacturer.

In the second step, the acceptor solution is pipetted into the acceptor vial. Typically, the volume of the acceptor is equivalent to about 50% of the vial volume. When using smaller volumes, the contact between the acceptor and the liquid membrane is markedly reduced, while completely filled vials counteract the agitation. The acceptor vial is then capped with the support membrane union (containing the support membrane).

In the third step, a microliter volume of membrane solvent is aspirated into a micropipette and subsequently dispensed onto the surface of the support membrane. The solvent droplet should be placed in the centre of the support membrane, and the pipette tip should not touch the surface of the support membrane. The membrane solvent diffuses into the entire pore volume of the support membrane after 30–60 s, and the liquid membrane is ready to use. The optimal volume depends on the type of membrane solvent; with NPOE, it is typically 8–9 µL with porous polypropylene membranes of 9 mm diameter and thickness of 110–170 µm. The optimal volume of membrane solvent can be determined by loading support membranes with different volumes of solvent and visually inspecting if the membrane is covered with membrane solvent, without excess solvent observed on the surface of the support membrane. Although the SLM solvent currently is immobilized immediately prior to extraction, it may theoretically be pre‐immobilized by the manufacturer in the future. Alternatively, dry membranes such as polymer inclusion membranes could also be used in this manner.

In the fourth step, the sample, sample diluent and internal standard are pipetted into the sample vial. The total volume of the resulting sample solution is typically equal to 50% of the total vial volume, for the same reasons as discussed for the acceptor. For the final assembly, the acceptor vial is secured onto the sample vial via the support membrane union.

Extraction cells are then loaded into the 10‐position holder, making sure that the extraction cell is aligned correctly in the electric circuit. In the extraction of protonated bases (cations), the acceptor vial is aligned with the cathode. To extract deprotonated acids (anions), the polarity is reversed. The lid is secured on top, agitation is initiated and the extraction potential is turned on. The extraction current is followed closely during operation, to assure system integrity. After the extraction, the acceptor vials are collected with the support membrane unions immediately removed. Acceptor vials should not be stored in contact with the liquid membranes, due to the risk of back‐extraction.

## 6 EME‐SPECIFIC CONSIDERATIONS

EME is a three‐phase extraction system driven by an electric field, and the operator should be aware of a couple of issues inherently connected to this principle.

### 6.1 Current

The extraction cell is an electrolytic cell. For extraction of bases, the sample vial acts as the anode, and the acceptor vial acts as the cathode. For the extraction of acids, the extraction potential is reversed. Once the extraction potential is turned on, a flux of analyte ions, sample diluent ions and sample matrix ions is established across the liquid membrane, and an electric current passes through the system (extraction current). Due to this current, electrolysis occurs, and water is oxidized in the anodic vial (Equation 1) and reduced in the cathodic vial (Equation 2):

(1) $$Anode:\;\;\;\;\;\;H_{2}O\left(l\right)\to 2H^{+}\left(\mathrm{aq}\right)+\frac{1}{2}O_{2}\left(g\right)+2e^{-}$$

(2) $$Cathode:\;\;\;\;\;\;2H^{+}\left(\mathrm{aq}\right)+2e^{-}\to H_{2}\left(g\right)$$

As illustrated by the equations, two fundamental problems arise from electrolysis.^27^, ^28^ First, gas is produced in both compartments, and if not controlled, pressure may increase because the system is closed. Increased pressure may challenge the stability of the liquid membrane. Second, pH may change on both sides of the liquid membrane. For extraction of basic analytes, pH increases in the acceptor, and this may be followed by analyte deprotonation and back‐extraction into the liquid membrane. High extraction current increases gas formation and pH changes and affects recoveries and precision negatively. The extraction current depends on the electric resistance of the liquid membrane and the sample matrix, and it is controlled by the chemical properties of the liquid membrane and the extraction potential. In general, the current is higher during extraction from biological samples as compared to aqueous buffers. Therefore, method development should include experiments from real samples.

**FIGURE 5:**
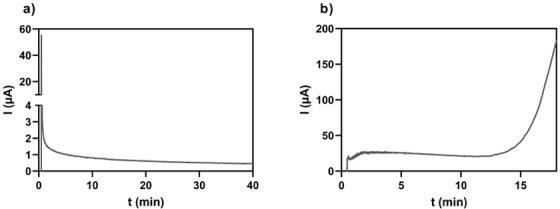
Recorded extraction currents in stable (a) and unstable (b) electromembrane extraction (EME) systems. (a) Liquid membrane = 2‐nitrophenyl octyl ether (NPOE), extraction potential = 50 V. (b) Liquid membrane = 6‐methylcoumarin:thymol (1:2, molar ratio) with 2% di(2‐ethylhexyl) phosphate (DEHP), extraction potential = 10 V. The sample was acidified serum, and the acceptor was 130 mM formic acid in both extractions.

The extraction current is recorded during EME, and it is a useful diagnostic tool to evaluate the integrity of the liquid membrane and thereby the system stability. In general, the extraction current in a stable EME system is constant or decreasing as a function of time and should be kept at <50 µA per extraction cell to minimize the effects of electrolysis. Figure 5 gives an example of extraction current monitored in a stable system with NPOE (a) as the liquid membrane, and in a more unstable system with a mixture of 6‐methyl‐coumarin, thymol and DEHP as the liquid membrane (b). A typical extraction current profile of a stable system is characterized by an initial rapid increase in current, followed by a gradual decrease and a final stabilization. Fundamentally, the liquid membrane behaves as a parallel coupling of a resistor and a capacitor. The initial peak current (often >50 µA) is due to the charging of the liquid membrane (capacitor) and is not considered when evaluating system stability.

Unstable systems are characterized by an increase in the extraction current as a function of time (Figure 5b). In unstable systems, the extraction current often exceeds the recommended limit of 50 µA. The membrane solvent leaks to the sample solution and acceptor, and water gradually penetrates the liquid membrane. The properties of the liquid membrane change gradually, the electric resistance is decreasing and the system may suffer from Joule heating. Unstable systems occur more frequently in the extraction of hydrophilic compounds with more hydrophilic liquid membranes or with the addition of ionic carriers (e.g. DEHP).^4^, ^29^ They must be operated with lower extraction potential and may not be applicable for very long extractions (>45 min).

### 6.2 Extraction efficiency

The extraction recovery (%ER) is defined as the degree of analyte transfer into the acceptor, and is calculated from the following equation:

(3) $$\%\mathrm{ER}=\frac{n_{a_{\mathrm{final}}}}{n_{s_{\mathrm{initial}}}}\times 100\%=\frac{C_{a_{\mathrm{final}}}\times V_{a}}{C_{s_{\mathrm{initial}}}\times V_{s}}\times 100\%$$

where $n_{a_{\mathrm{final}}}$ and $n_{s_{\mathrm{initial}}}$ are the number of moles of analyte in the acceptor after extraction and the number of moles of analyte in the sample at *t* = 0, respectively. Correspondingly, $C_{a_{\mathrm{final}}}$ and $C_{s_{\mathrm{initial}}}$ are the analyte concentrations in the acceptor after extraction and in the sample at *t* = 0. *V*_a_ and *V*_s_ denote the volume of the acceptor and sample, respectively.

In most EME systems of high efficiency, the sample solution is depleted from the analyte. Despite this, recoveries are often less than 100% because a small fraction of analyte molecules may be trapped in the liquid membrane. Therefore, recovery ≥85% may be considered exhaustive. Under such conditions, EME normally provides linearity, precision and accuracy comparable to classical sample preparation methods such as protein precipitation and solid‐phase extraction. However, exhaustive extraction is no prerequisite for high data quality, as this is normally obtained for compounds extracted with recoveries >40%. For compounds extracted less than 40%, precision is often influenced by the fact that a large fraction of analyte molecules are left in the sample solution or trapped in the liquid membrane after extraction.

### 6.3 Electrochemical stability

In some cases, analytes extracted using EME may undergo degradation due to instability at the pH conditions of the system or due to the electric field. This can lead to low extraction recovery and poor precision and accuracy. If pH is an issue, the operator should investigate extraction at neutral pH. In this case, neutral pH should be controlled by use of suitable buffer solutions rather than pure water, as the latter is susceptible to pH change from electrolysis effects. If the analyte is not charged or only partly charged at physiological pH, extractions by LPME of the neutral species may alternatively be performed. Electrochemical instability may be addressed by the addition of an antioxidant, such as ascorbic acid, to the sample solution and to the acceptor.^9^

## 7 APPLICATIONS, TWO EXAMPLES

### 7.1 Extraction of psychoactive drugs

A representative example of conductive vial EME was published by Skaalvik et al. in 2021.^30^ The work aimed to evaluate the performance of EME in a routine hospital laboratory and to compare it with existing routine sample preparation methods based on protein‐precipitation and phospholipid removal. Samples were human serum spiked with 12 psychoactive drug substances and corresponding metabolites with basic functionality. Prior to extraction, 100 µL serum was pipetted into the sample vial, and 25 µL internal standard and 175 µL 0.1% v/v formic acid were added, the latter to acidify the sample and ensure protonation of the basic analytes. NPOE of 9 µL was used to prepare the liquid membrane, whereas 300 µL 0.1% v/v formic acid was used as an LC–MS friendly acceptor. After the assembly of the extraction cells, EME was performed at 50 V for 15 min with horizontal agitation at 875 rpm to ensure sufficient convection. Subsequently, the acceptors were analysed by UHPLC–MS/MS. The final method was subjected to full validation and was compliant with FDA guidelines for bioanalytical methods. Extraction recoveries were 75%–117% (median 88%), matrix effects (ion suppression) were 94%–104% (100% corresponds to no matrix), intra‐ and inter‐day precision <6% relative standard deviation (RSD) and extracts were determined to be free of phospholipids. The results from analysing 30 real patient samples were also in agreement with the routine method. The current method may serve as a generic EME system for basic analytes with 2.0 < log *P* < 6.0.

### 7.2 Extraction of methotrexate

In another example, Hay et al. developed a method for the extraction of methotrexate (MTX, chemotherapy agent), and its metabolites 7‐hydroxymethotrexate (7‐OH‐MTX) and 4‐amino‐4‐deoxy‐*N*(10)‐methylpteroic acid (DAMPA) from human plasma.^31^ MTX, 7‐OH‐MTX and DAMPA are challenging to extract because they are hydrophilic and zwitterionic substances. In principle, the substances can be extracted as either cations or anions. After testing a broad range of experimental conditions, the deep eutectic solvent comprising 6‐methylcoumarin and thymol in 1:2 molar ratio, with 0.5% w/w DEHP was selected as liquid membrane. MTX, 7‐OH‐MTX and DAMPA were extracted as cations. The authors optimized the operational parameters to maximize sensitivity in the subsequent LC–MS analysis. Highest sensitivity was obtained by using a sample volume of 500 µL, acceptor volume of 200 µL, 900 rpm agitation, 10 V and 30‐min extraction time. The sample solution was prepared by adding 250 µL dilute phosphoric acid and internal standard to 250 µL plasma, to adjust pH to 2.4 where MTX, 7‐OH‐MTX and DAMPA are cationic. Under these conditions, extraction recoveries were 23%–53%, calibration curves were linear (*R* ^2^ ≥ 0.9952), accuracy was in the range 98%–121%, RSD was 2.8%–27.6% and matrix effects were low at 103%–111%. The current method may serve as a generic EME system for zwitterionic analytes.

## 8 CONCLUDING REMARKS AND OUTLOOK

Conductive vial EME will be commercially available in a short time, and we expect this to increase the interest for EME. A major incentive for further development and future use of EME may be to perform green sample preparation. Next‐generation analytical scientists will be very focused on sustainability and green aspects. Another incentive may be to take advantage of the unique selectivity of EME, which to large extent is controlled by the external electric field. No other sample preparation or extraction technique is, to our knowledge, driven and controlled by an electric field. We expect conventional sample preparation procedures to be replaced by EME to some extent, but most likely EME will be more important in areas where the use of traditional techniques is complicated. This may be in cases where the sample matrix is highly complex, such as with tissue samples^32^; or in cases where the analytes are very complex molecules, such as with proteins and peptides. Finally, unlike traditional extraction techniques such as liquid–liquid extraction and solid‐phase extraction, EME is perfectly suited for implementation in microchip technologies.^33^ For all future work with EME, the basic understanding presented in this tutorial is important, to avoid pitfalls and to develop robust solutions.

## AUTHOR CONTRIBUTIONS

*Conceptualization; writing – original draft; writing – review; and editing; visualization*: Maria Schüller, Frederik André Hansen, Tonje Gottenberg Skaalvik. *Conceptualization; supervision; writing – original draft; writing – review and editing*: Stig Pedersen‐Bjergaard.

## CONFLICT OF INTEREST STATEMENT

The authors declare no conflicts of interest.

## Data Availability

Data sharing is not applicable to this article as no new data were created or analysed in this study.
